# Comparing reintroduction strategies for the endangered San Francisco gartersnake (*Thamnophis sirtalis tetrataenia*) using demographic models

**DOI:** 10.1371/journal.pone.0292379

**Published:** 2023-10-05

**Authors:** Jonathan P. Rose, Richard Kim, Elliot J. Schoenig, Patrick C. Lien, Brian J. Halstead

**Affiliations:** 1 U.S. Geological Survey, Western Ecological Research Center, Santa Cruz, California, United States of America; 2 U.S. Geological Survey, Western Ecological Research Center, Dixon, California, United States of America; PLOS ONE, UNITED KINGDOM

## Abstract

For endangered species persisting in a few populations, reintroductions to unoccupied habitat are a popular conservation action to increase viability in the long term. Identifying the reintroduction strategy that is most likely to result in viable founder and donor populations is essential to optimally use resources available for conservation. The San Francisco gartersnake (*Thamnophis sirtalis tetrataenia*) is an endangered sub-species that persists in a small number of populations in a highly urbanized region of California. Most of the extant populations of San Francisco gartersnakes have low adult abundance and effective population size, heightening the need for establishment of more populations for insurance against the risk of extinction. We used simulations from demographic models to project the probability of quasi-extinction for reintroduced populations of San Francisco gartersnakes based on the release of neonate, juvenile, adult, or mixed-age propagules. Our simulation results indicated that the release of head-started juveniles resulted in the greatest viability of reintroduced populations, and that releases would need to continue for at least 15 years to ensure a low probability of quasi-extinction. Releasing captive-bred juvenile snakes would also have less effect on the viability of the donor population, compared to strategies that require more adult snakes to be removed from the donor population for translocation. Our models focus on snake demography, but the genetic makeup of donor, captive, and reintroduced populations will also be a major concern for any proposed reintroduction plan. This study demonstrates how modeling can be used to inform reintroduction strategies for highly imperiled species.

## Introduction

For species with narrow ranges surrounded by urban and suburban development, the number and connectivity of extant populations is an important predictor of viability [1]. Recovery plans for species listed under the United States Endangered Species Act (ESA) often specify targets for the number and abundance of populations for recovery to be achieved [2]. Establishing several viable populations can be a crucial step in ensuring a taxon has the resiliency, redundancy, and representation for persistence in the face of future change [3]. If populations are connected through suitable dispersal habitat, genetic diversity can be maintained through the exchange of individuals [4] and demographic rescue can prevent extirpation [5]. When only a few isolated populations remain, reintroduction of individuals to unoccupied but suitable habitat is frequently attempted to decrease extinction risk for threatened and endangered species.

With limited resources available and high stakes for imperiled species, research into what distinguishes successful reintroductions from failures is prevalent [6]. Successful reintroductions have been documented for plants [7], invertebrates [8], and vertebrates [9–11]. Conversely, some reintroduction efforts have failed to establish viable populations despite substantial investment [12]. The success or failure of a reintroduction can be influenced by the size of the propagule [7, 9], life stage(s) released [13], the duration of the reintroduction effort [11], or whether survival and reproduction of reintroduced individuals is concordant with predictions based on wild populations [14, 15]. Although much has been learned by reviewing outcomes of past reintroductions, reintroduction biology can benefit from studies that explicitly seek to answer questions defined *a priori* to support decision makers [16]. Using models to make testable predictions about alternative hypotheses could be a fruitful path forward for reintroduction biology [17].

Demographic population models are a powerful tool for evaluating reintroduction strategies and predicting which approaches are most likely to produce a viable population [18]. Simulating future population trajectories with a demographic model allows for comparing reintroduction strategies and assessing trade-offs [19, 20]. One major decision is which life stage(s) to release when founding a new population [21]. One option is to “head-start” juveniles in captivity to reach a larger size and reduce the risk of mortality. Head-starting is frequently employed for reintroductions [22] because it reduces costs of caring for animals long-term [23], and ameliorates potential fitness costs of being raised in captivity [15, 24]. Alternatively, translocation of adults directly from donor populations could be more effective [25] because of higher post-release survival in wild-caught animals compared to captive-born individuals [26] and because adults can quickly reproduce and establish the new population. The effect of reintroduction strategy on the viability of the donor population also deserves consideration, and might depend on the life stage removed [27]. Given the number of variables involved, it is difficult to experimentally evaluate reintroduction strategies for rare taxa with small populations. Modeling efforts that simulate the expected outcomes under different reintroduction strategies are valuable for these rare taxa.

The San Francisco gartersnake (*Thamnophis sirtalis tetrataenia*) is a subspecies of the common gartersnake that is listed as endangered under the U.S. ESA [28] and California ESA [29] and an exemplar of a taxon persisting in a few isolated populations. The San Francisco gartersnake is endemic to the San Francisco peninsula, a highly urbanized region of California, USA, and persists in just 10 known extant population complexes [30], most of which have low adult abundance and genetic diversity [31]. The recovery plan for the San Francisco gartersnake requires that 10 populations have an abundance of at least 200 adult snakes and a 1:1 sex ratio for a period of 15 years for the subspecies to be considered recovered [32]. Given the current distribution and abundance of known extant populations [31, 33, 34], this goal would be met if existing populations increased in abundance and new populations were established.

To conserve this endangered subspecies, the U.S. Fish and Wildlife Service is exploring the feasibility of a captive-breeding and reintroduction program for the San Francisco gartersnake [30]. The objectives of the captive-breeding and reintroduction program are to 1) Increase the number of extant San Francisco gartersnake populations by reintroducing the species to suitable but unoccupied habitat within the historical range, 2) Ensure that new populations establish and persist in the long-term (≥ 15 years), and 3) Minimize the impact on existing populations used as donors for the reintroduction program. This study seeks to address questions relevant to objectives 2 and 3, including: Which life stages should be released? How many snakes should be released in each propagule? How long do releases need to continue to have a high probability of persistence in the next 30 years? We built a size-structured demographic model for the San Francisco gartersnake to identify which vital rate transitions contribute most to population growth and simulate how reintroduction strategies influence the viability of reintroduced and donor populations. This study demonstrates how demographic models can be used to identify preferred reintroduction strategies and generate predictions of population growth that can be compared to observed population trajectories in reintroduced populations.

## Methods

### Data sources

The demographic data used to construct the integral projection model (IPM) came from three primary sources (Table 1): 1) capture-mark-recapture data used to estimate survival and growth rates of San Francisco gartersnakes [35], 2) ultrasound scans of female San Francisco gartersnakes to estimate fecundity and the probability a female is gravid [36], and 3) vital rate estimates from other gartersnakes and natricine snakes reported in the literature [37–42]. Field research on San Francisco gartersnakes was performed under California Scientific Collecting Permit SCP-10779 and USFWS permit TE-157216, and the Animal Care and Use protocol WERC-2014-01.

**Table 1:** Functions used to build the San Francisco gartersnake (*Thamnophis sirtalis tetrataenia*) integral projection model and the sources of the data used to fit those functions. A ‘-’ indicates the summary statistic is not applicable to that parameter. The smooth spline function for survival, f′(x), is a nonlinear function composed of multiple parameters and cannot be characterized by a mean and standard deviation (SD).

Function	Equation	Parameter	Description	Mean	SD	Source
Fecundity	7	π	Intercept of size-fecundity relationship	-0.089	0.396	[36]
	7	ω	Slope of size-fecundity relationship	4.25E-03	5.90E-04	[36]
	9	*s*_*nn*_	Neonate survival to juvenile age	0.1–0.4	-	[37–39]
	7	*s*_*emb*_	Survival rate from embryo to parturition	0.82	-	[40]
	7	*p*_*f*_	Proportion of embryos that are female	0.5	-	[34, 41, 42]
Reproduction	6	γ	Intercept of size-reproductive status relationship	-6.88	2.37	[36]
	6	β	Slope of size-reproductive status relationship	0.0131	3.93E-03	[36]
	6	SVL_min_	Minimum size at sexual maturity (mm)	450	-	[36]
	8	μ_*rec*_	Mean size at recruitment (mm)	338.9	-	[35, 36]
	8	σ_*rec*_	SD of size at recruitment	46.36	-	[35, 36]
Growth	3	*k*	von Bertalanffy growth coefficient	313.7	40.8	[35]
	3	L_∞_	Asymptotic snout-vent length (mm)	744.5	17.3	[35]
	4	σ_*x*_	SD of growth around expected value	23.3	2.1	[35]
	4	σ_*g*,*t*_	SD of annual random effect on growth	1.2	0.1	[35]
Survival	5	α	Intercept of annual survival	0.706	0.524	[35]
	5	f′(x)	Smooth spline function for size-survival	-	-	[35]
	-	σ_*s*,*t*_	SD of annual random effect on survival	1.186	0.483	[35]

### Integral projection model

**Fig 1:**
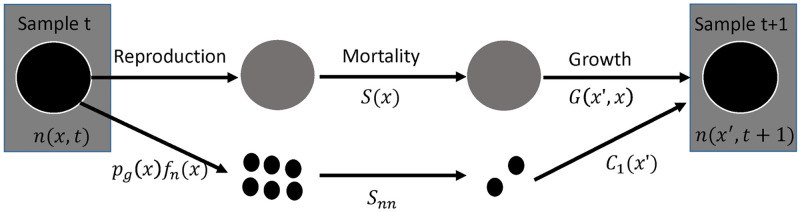
Life-cycle diagram for the San Francisco Gartersnake (*Thamnophis sirtalis tetrataenia*) integral projection model. The model represents a pre-reproductive census where reproduction takes place after the population is censused and recruits are added to the population at approximately one year old.

The IPM is a female-based model with an annual time-step based on a pre-reproductive census; surveys took place in the spring from March to June [35] and parturition in San Francisco gartersnakes occurs in the summer [34]. Parturition takes place shortly after the population is censused, and therefore new recruits join the population when they are almost 1 year old (Fig 1). Snake snout-vent length (SVL, in mm) is the state variable that determines the growth, survival rate, fecundity, and reproductive status of snakes. The IPM models how the size distribution at time *t*+1, *n*(*x*′,*t*+1) is a function of the size distribution at time *t*, *n*(*x*,*t*) and the kernel, *K*(*x*′,*x*), which is a demographic model of how the size distribution *x* at time *t* produces size distribution *x*′ at time *t*+1. The kernel is composed of growth, *G*(*x*’,*x*), survival, *S*(*x*), and reproduction components, *F*(*x*′,*x*) (Eqs 1 and 2). The sizes included in the IPM (Ω) range from a minimum of 100 mm SVL (75 mm shorter than the smallest snake in our sample, [35]; approximately 60 mm shorter than average neonate length reported by Barry [43]) to a maximum of 900 mm SVL (85 mm SVL longer than the largest female in our sample, [35]). We tested whether these size limits were sufficient to prevent eviction of individuals from the model [44]; we found no evidence of eviction affecting estimates of λ (Δλ < 2 x 10^−10^).


$$n\left(x^{'},t+1\right)=\int_{\Omega }^{}K\left(x^{'},x\right)n\left(x,t\right)dx$$ (1)

$$K\left(x^{'},x\right)=G\left(x^{'},x\right)S\left(x\right)+F(x^{'},x)$$ (2)

**Fig 2:**
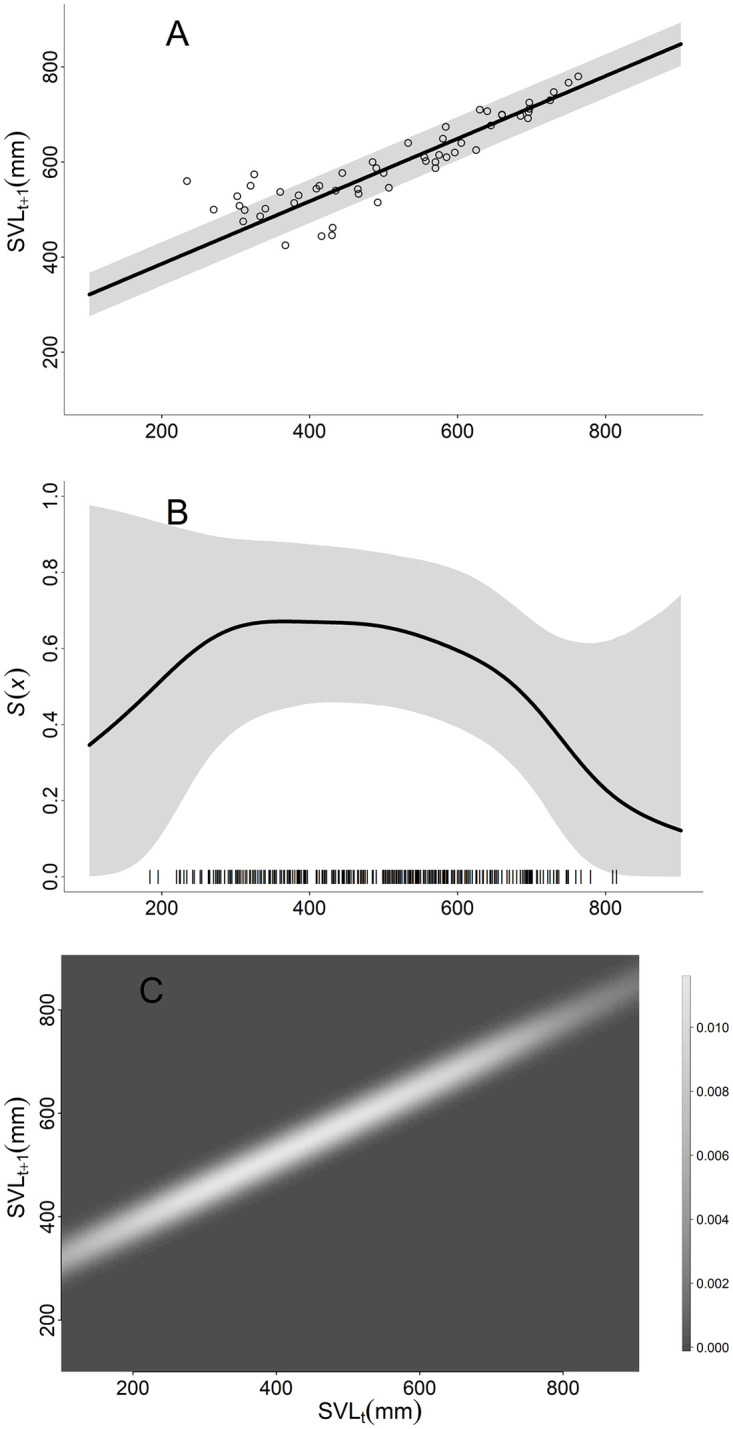
Growth and survival functions. The A) growth and B) survival functions and C) survival-growth kernel used to build the San Francisco gartersnake (*Thamnophis sirtalis tetrataenia*) integral projection model. Vertical tick marks on the x-axis in B represent the snout-vent length (SVL) of marked snakes used to estimate survival. In C, the shade of gray indicates the probability density of survival and growth from x at time *t* to x′ at time *t*+1, lighter colors indicate higher probability density.

We defined survival and growth functions based on capture-mark-recapture modeling in [35]. Growth follows a von Bertalanffy function in which individual growth slows with age as snakes approach an asymptotic SVL (*L*_∞_) based on the growth coefficient parameter *k* (Eq 3; Fig 2). The distribution of size *x*′ at time *t*+1 is based on a normal distribution centered on the expected size, *μ*_*g*_(*x*) and the standard deviation of expected size, *σ*_*x*_ (Eq 4). Survival is a non-linear spline function of SVL, *f*′(*x*), in which survival is highest for snakes between 300–550 mm SVL and declines for snakes between 550 mm and 700 mm SVL (Eq 5, Fig 2). The annual survival rate is highly uncertain for snakes < 300 mm and > 700 mm SVL [35], necessitating an approach that accounts for parametric uncertainty (see *Bayesian IPM* below). Because recruits enter the population at approximately 1 year old, the survival rate of snakes during the first year (i.e., < 300 mm SVL) is modeled in the fecundity function (see below).


$$\mu_{g}\left(x\right)=L_{t}+\left(L_{\infty }-L_{t}\right)\times \left(1-exp\left(\frac{k}{L_{\infty }}\times \Delta t\right)\right)$$ (3)

$$G\left(x\prime ,x\right)\;\sim \;Normal\left(\mu_{g}\left(x\right),\sigma_{x}\right)$$ (4)

$$S\left(x\right)=\alpha +f^{'}\left(x\right)$$ (5)

The fecundity and reproductive status of female snakes was determined by ultrasound in the field during March–May from 2019 to 2022 using a SonoSite MicroMaxx portable ultrasound and a SonoSite SLA 13–6 MHz linear transducer (FUJIFILM SonoSite, Inc., Bothell, WA, USA; [36]). The abdomen of female snakes was covered with a lubricating jelly and all enlarged eggs/follicles and embryos were counted as the abdomen was scanned. Of 50 gravid females, the mean embryo count was 15.3 (range = 7–27 embryos) [36]. Females that were determined to be too small or thin to be gravid were not given an ultrasound. We fit a Bayesian logistic regression model with female SVL as the covariate and reproductive status (*p*_*g*_; 1 if gravid, 0 if not) as the response (Eq 6).


$$logit\left(p_{g}\left(x\right)\right)=\gamma +\;\beta \left(x\right)\times I(x\;\geq \;{SVL}_{min})$$ (6)

$$fec\left(x\right)=p_{f}\times s_{emb}\times exp\left(\pi +\;\omega (x)\right)$$ (7)

$$C_{1}\left(x\prime \right)\;\sim \;Normal\left(\mu_{rec},\sigma_{rec}^{2}\right)$$ (8)

$$F\left(x\prime ,x\right)=p_{g}\left(x\right)\times fec\left(x\right)\times s_{nn}\times C_{1}\left(x\prime \right)$$ (9)

**Fig 3:**
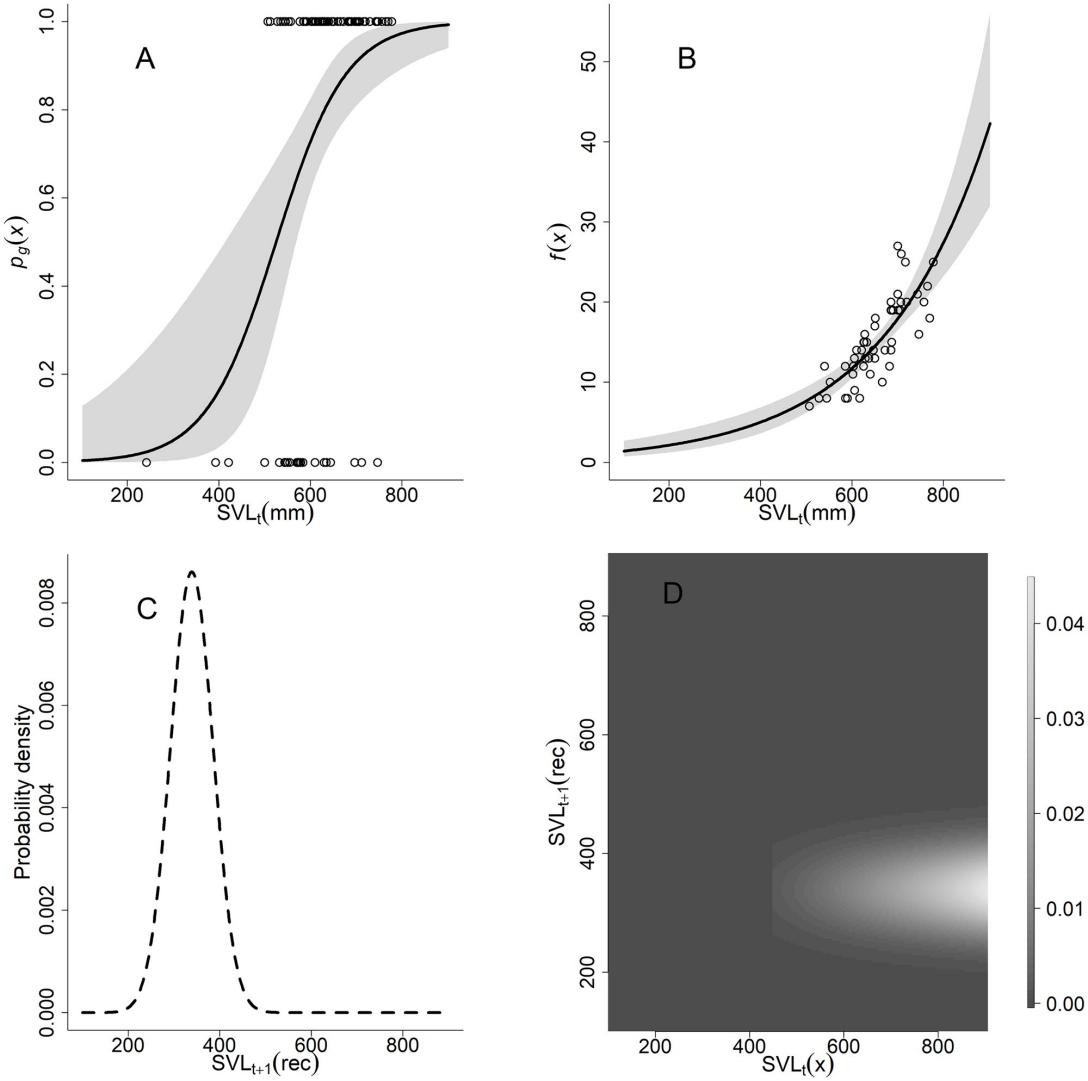
Reproduction functions. The A) probability of reproduction and B) fecundity functions, C) recruit size distribution, and D) reproduction kernel of the San Francisco gartersnake (*Thamnophis sirtalis tetrataenia*) integral projection model. In A, circles at the top of the plot (y = 1) represent snout-vent lengths (SVL) or females that were gravid, circles at the bottom of the plot (y = 0) represent SVLs of females that were not gravid. The mean fit line and 95% credible interval (gray shading) were estimated using a Bayesian implementation of logistic regression. In B, circles are observed embryo counts of examined females; the mean fit line and 95% credible interval were estimated using a Bayesian implementation of a Poisson regression. In D, the shade of gray represents the density of the contribution of a snake at size x at time *t* on the x-axis to the recruit size at time *t*+1 on the y-axis.

Because female San Francisco gartersnakes are unlikely to be sexually mature until they reach at least 2 years of age, we set a minimum size threshold (SVL_min_) of 450 mm SVL to reproduce; the indicator function I(x ≥ SVL_min_) has a value of 1 if SVL is greater than or equal to the minimum SVL at sexual maturity and 0 otherwise. Snake fecundity as a function of female SVL, *fec*(*x*), was estimated using a Poisson regression (Fig 3; Eq 7). Stillbirth and undeveloped embryos have been observed in litters of other gartersnake species [45, 46]. Therefore, embryos or eggs counted in gravid females during the spring might not survive and develop into live neonates. The fecundity function included a parameter for embryo survival (*s*_emb_) based on data from giant gartersnake litters born in captivity [40] (Table 1). We assumed a 1:1 sex ratio and the embryo count was multiplied by *p*_*f*_ = 0.5 to include only female offspring. Equation 8 describes the size distribution of approximately one-year-old recruits *C*_*1*_(*x*′) as a normal distribution with a mean (*μ*_*rec*_) and variance (*σ*^2^_*rec*_) based on the projected growth from size at birth (mean = 165 mm SVL, SD = 10 mm [43]) to size at 1 year old [35]. Because recruits are added to the population just before they reach one-year-old, neonate survival during the first year (*s*_*nn*_) is part of the reproduction component (Eq 9) instead of the survival function. Capturing neonate San Francisco gartersnakes is difficult and we lack data on the survival rate during the first year [35]. For all IPM analyses, we built models with a range of plausible values for neonate survival to one year of age (0.10, 0.20, 0.30, and 0.40) based on estimates from studies of other natricine snakes [37–39, 47].

### Elasticity analysis

To determine which life stage transitions contribute the most to San Francisco gartersnake population growth, we calculated the elasticity of λ to changes in vital rate functions. Elasticity is the proportional sensitivity; it scales the effect of perturbing the IPM kernel by the fractional change in that transition, therefore discounting the importance of implausible transitions (e.g., from recruit to large reproductive adult size in one year). We quantified the elasticity of λ to changes in vital rate functions by calculating how perturbing a vital rate function (e.g., Eq 5) affects λ for all sizes within the domain of the IPM. Elasticities were calculated following the methods of [48] for the range of neonate survival values.

To incorporate parametric uncertainty into analysis of the IPM and estimates of λ we constructed a Bayesian IPM following [49]. We drew 10,000 samples from the posterior distributions of Bayesian regression models underlying the survival, growth, fecundity, and reproductive status functions [35] and constructed IPM kernels using a different set of parameter estimates for each. We then calculated λ for each IPM kernel and summarized the estimate of λ by the mean and 2.5^th^ and 97.5^th^ percentiles for the distribution of λ values. We also constructed stochastic IPMs in which the growth rate (*k*) and mean survival rate varied among years to calculate the log stochastic population growth rate, log(λ_s_) following [48].

### Population simulations

To model the effectiveness of reintroduction strategies while accounting for demographic stochasticity and transient population dynamics, we ran individual-based simulations using growth, survival, and reproduction functions from the IPM. We simulated the dynamics of reintroduced populations for 30 years and incorporated environmental stochasticity in the form of annually varying mean growth rates (*k*) and survival rates (*S*) that were drawn for each year of a simulation using the annual random effects estimated from capture-mark-recapture data for San Francisco gartersnakes (Table 1; [35]). We ran 1000 replicated simulations using a different sample from the posterior distributions of the growth, survival, fecundity, and reproduction functions for each simulation to incorporate parametric uncertainty [50].

Each simulation began with a founder population of snakes with SVLs drawn from a normal distribution based on the reintroduction strategy: A) neonates released shortly after birth (mean = 165 mm SVL, SD = 10), B) head-started snakes raised in captivity and released at one-year-old (mean = 338.9 mm SVL, SD = 46.4), C) adults translocated directly from the donor population to the reintroduced population (mean = 650 mm SVL, SD = 50), or D) mixed size distributions of neonates, juveniles, and adults released. Further, we modeled reintroductions in which snakes were released annually for the first 1, 5, 10, 15, or 20 years of the 30-year period (Table 2). We did not model a strategy based on the creation of a self-sustaining captive breeding population for release because limiting the number of generations in captivity is desirable to reduce the effects of genetic adaptation to captivity [24]. Finally, for each of the four reintroduction strategy x five release duration combinations, we ran models with neonate survival set to 0.10, 0.20, 0.30, and 0.40 to reflect the uncertainty in this parameter, and simulated the outcome if 3, 5, or 10 adult females were used to produce the release cohorts, resulting in a total of 240 simulated scenarios (Table 2). To prevent simulated populations from reaching unrealistic abundances, we set a maximum abundance of 1000 female snakes and if the population increased above this maximum, 1000 individuals were randomly sampled to define the population state at the next time step. We calculated the probability of quasi-extinction (*N* < 5 female snakes) during the 30-year simulation period for each scenario. As with the IPM, we only modeled the fate of female snakes, and we assume that an equal number of male snakes are released in all scenarios. Both male and female *T*. *sirtalis* can mate with multiple partners [51, 52], and males are unlikely to be a limiting resource preventing reproduction.

**Table 2:** Reintroduction scenarios for San Francisco gartersnakes (*Thamnophis sirtalis tetrataenia*) that vary in the age of individuals released to initiate the new population and the number of individuals released during each event. Each strategy was repeated with releases occurring annually for 1, 5, 10, 15, or 20 years. Adults req. = The number of adults required to be kept in captivity to produce the necessary propagules of neonates and juveniles to release (strategies A, B, and D) or to translocate adults directly from the donor population (strategy C).

Strategy	Age released	Adults req.	Number per release
A	Neonates	10	48
B	Juveniles	10	36
C	Adults	10	10
D	Mixed	10	41
A	Neonates	5	24
B	Juveniles	5	18
C	Adults	5	5
D	Mixed	5	21
A	Neonates	3	14
B	Juveniles	3	11
C	Adults	3	3
D	Mixed	3	11

Because captive-reared animals might experience lower survival when released into the wild than wild-born animals [53], it is possible that simulations based on releasing captive-reared juveniles provide overly optimistic projections of population growth. To model the effect of reduced survivorship after captivity, we ran additional simulations for strategy B in which captive-reared juveniles experience reduced survival in their first year post-release into the wild, following [20]. We ran simulations in which juvenile survival in the first year after release was varied from 10% to 90% (in increments of 10%) of expected survival for wild-born juveniles, and compared the probability of quasi-extinction (*N* < 5) to strategy A, the release of neonate snakes immediately after birth.

We also modeled the effect of removing adult females on the viability of donor populations. We set initial population sizes for the donor populations to 100 or 650 females based on abundance estimates from the two largest known San Francisco gartersnake populations [31]. We then removed adult snakes (SVL ≥ 450 mm) from the donor populations matching the number of adults required to sustain reintroduction strategies based on releasing neonates or juveniles born in captivity (A or B), adults translocated directly from the donor population (C) or a mixed size-distribution of neonate, juvenile, and adult snakes (D). We set the annual survival rate of neonate snakes in the wild to 0.30 for all donor population simulations, which would result in a slowly growing population (λ ≈ 1.01) in a stochastic environment if no adults were removed. For the donor population, strategies A and B are equivalent (i.e., if 10 adults are removed, it does not matter if those adults produce captive-born neonates or juveniles for release), so we ran one set of simulations for the donor population representing strategy B.

We simulated donor populations for 30 years and ran 1000 replicated simulations to incorporate parametric uncertainty in growth, survival, fecundity, and reproduction. We set the maximum population size to 1000 individuals in the donor populations. Donor population simulations were based on removals necessary for the reintroduction strategy: B) a captive population of 10 adult females for breeding assuming 60% annual survival of adult females in captivity (10 adult females removed in first year, 4 adult females removed annually for years 2 to T, the end of reintroduction), C) annual translocations of 10 adult females from the donor population to the reintroduced population (10 adult females removed annually for years 1 to T), or D) 5 adult females translocated directly each year, and 5 adult females maintained in captivity to produce neonate/juvenile snakes (10 adults removed the first year, 7 adults removed annually for years 2 to T).

## Results

### Integral projection model

The mean population growth rate from the IPM predicts the population to decline rapidly when neonate survival is 0.10 (λ = 0.85), decline slowly when neonate survival is 0.20 (λ = 0.99) and increase rapidly when neonate survival is 0.30 (λ = 1.09) or 0.40 (λ = 1.17). The Bayesian IPM illustrates that there is uncertainty in estimates of λ because of parametric uncertainty, with credible intervals for λ overlapping 1 for all neonate survival scenarios (Table 3). Results from fitting a stochastic IPM where annual growth (*k*) and survival rates varied were qualitatively similar to the deterministic IPM. The estimated long-term log stochastic growth rate, log(λ_*s*_) represented slower growth than the asymptotic population growth rate from the deterministic IPM for all neonate survival values (Table 3).

**Table 3:** Estimated finite population growth rate (λ) from the Bayesian integral projection model (IPM) for the San Francisco gartersnake (*Thamnophis sirtalis tetrataenia*) and the log stochastic population growth rate (log λ_s_) from stochastic simulation of the IPM with different parameter values for the neonate survival rate. SD = standard deviation, 2.5% = 2.5^th^ percentile of the distribution of λ or log(λ_s_) values, 97.5% = 97.5^th^ percentile of the distribution of λ or log(λ_s_) values.

Neonate survival rate	Mean λ	SD λ	2.5% λ	97.5% λ	Mean log λ_s_	SD log λ_s_	2.5% log λ_s_	97.5% log λ_s_
0.10	0.846	0.107	0.636	1.053	-0.257	0.364	-1.143	0.204
0.20	0.988	0.119	0.761	1.218	-0.087	0.337	-0.906	0.336
0.30	1.092	0.129	0.843	1.337	0.017	0.325	-0.783	0.422
0.40	1.175	0.142	0.893	1.450	0.089	0.320	-0.688	0.487

**Fig 4:**
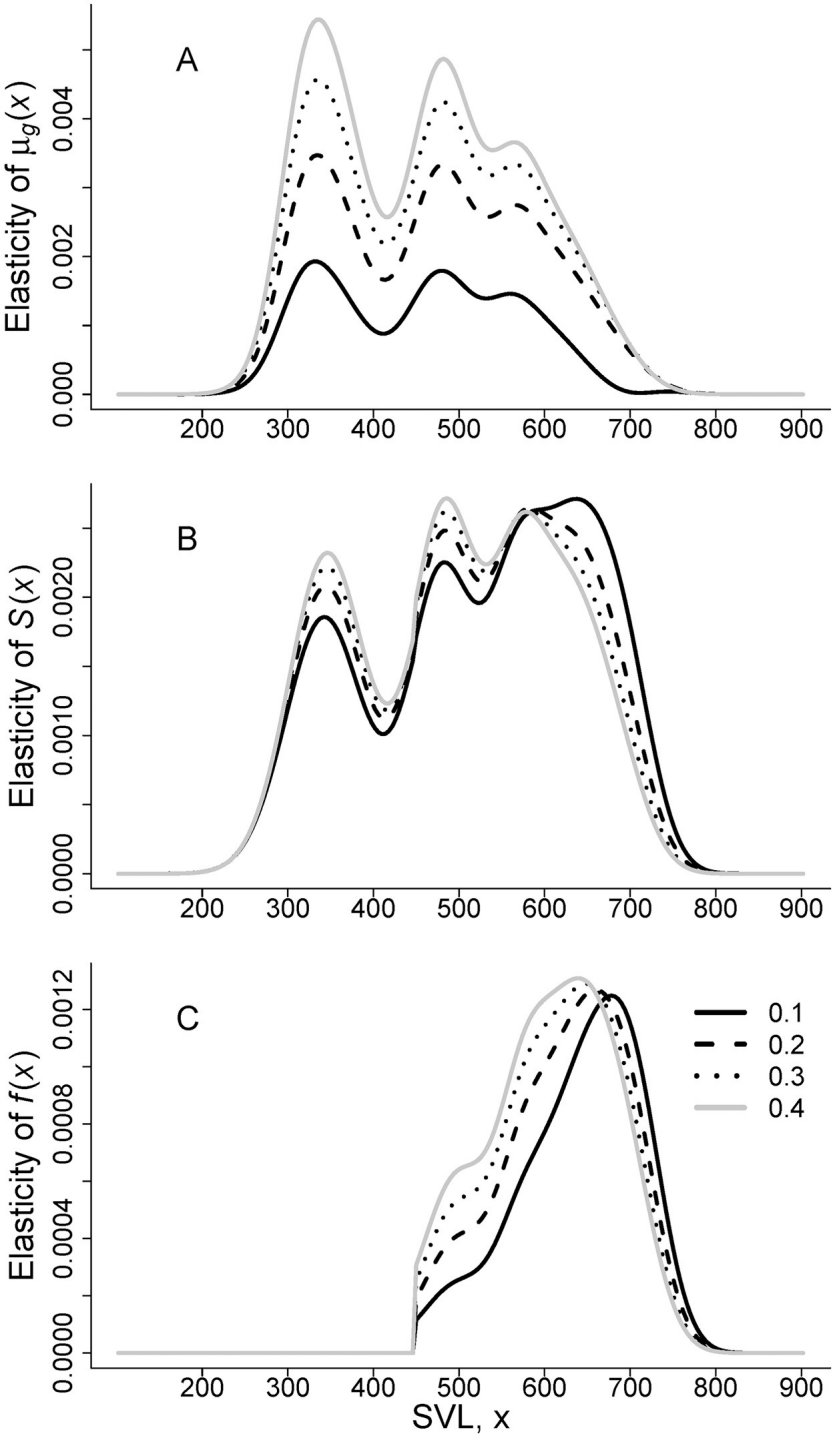
Elasticity of λ to vital rate functions for the San Francisco gartersnake (*Thamnophis sirtalis tetrataenia*). A) expected size in the next year, B) survival, C) fecundity. Color and pattern of line indicates the neonate survival rate to 1-year old (*s*_*nn*_): solid black line = 0.10, dashed black line = 0.20, dotted black line = 0.30, solid gray line = 0.40.

Changes to the survival and growth of female snakes have a greater influence on λ than changes to reproduction. The contribution of survival and growth to λ (0.79, 0.76, 0.74, 0.73 for neonate survival values of 0.10, 0.20, 0.30, and 0.40 respectively) was greater than the contribution of reproduction to λ (0.21, 0.24, 0.26, and 0.27) for all neonate survival scenarios, but the contribution of reproduction to λ increased as neonate survival increased from 0.10 to 0.40. The elasticity of λ to changes in the vital rate functions depended on the neonate survival rate. When neonate survival was 0.10, the survival and fecundity of the largest adult female snakes has the greatest proportional contribution to λ (Fig 4). As neonate survival increased from 0.10 to 0.40, the influence of the survival rates of 1-year old snakes and small adults (~ 500 mm SVL) on λ increased, indicating that survival of juvenile and young adult females was more important when neonates survived at a higher rate (Fig 4). Likewise, the snakes whose fecundity influenced λ the most shifted to smaller sizes as neonate survival increased, representing the greater contribution to reproduction of more abundant average-sized female snakes (600–650 mm SVL). The expected growth of 1-year-old snakes during their second year, and of snakes around 450–500 mm SVL had greater influence on λ than the growth of adult females > 600 mm SVL (Fig 4).

### Simulated reintroductions

**Fig 5:**
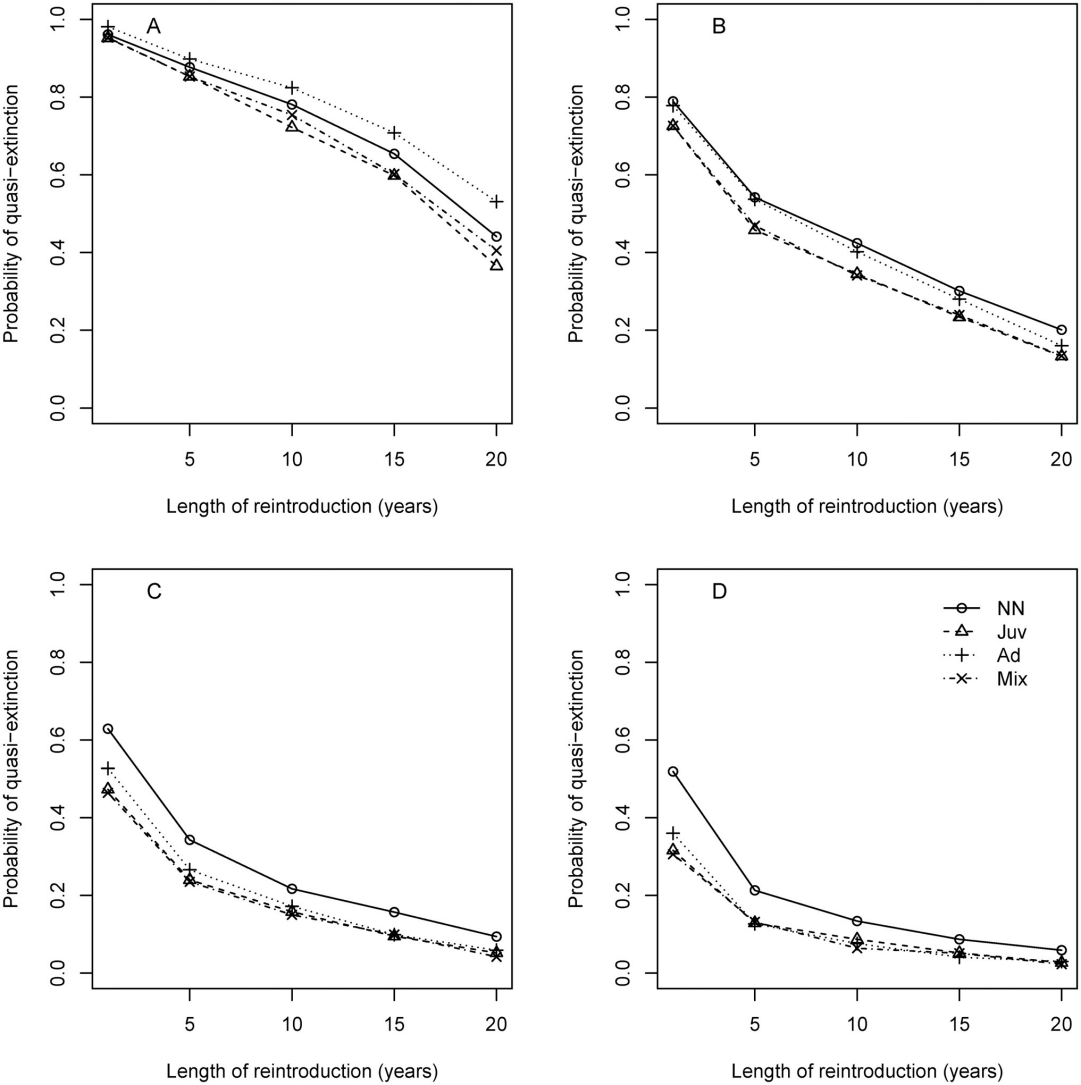
Probability of quasi-extinction (over a 30-year simulation) vs. the length of the reintroduction (the number of years in which snakes are released) for reintroduced populations of San Francisco gartersnake (*Thamnophis sirtalis tetrataenia*). Neonate survival rate in the wild set to A) 0.10, B) 0.20, C) 0.30, or D) 0.40. The four lines in each plot correspond to the life-stage released into the reintroduced population: neonates, juveniles, adults, or mixed age/size-distribution. For all scenarios, ten adult females are kept in captivity or released annually.

The number of adult snakes required (either for direct translocation or captive-breeding), reintroduction strategy, neonate survival rate in the wild, and the duration of the reintroduction effort all influenced the probability of quasi-extinction (*N* < 5) for reintroduced populations. Scenarios that required 10 adult females (Fig 5) resulted in lower quasi-extinction probabilities than scenarios requiring 3 or 5 adult females (S1 and S2 Figs), and hereinafter we focus on scenarios requiring 10 adult females. The duration of the reintroduction effort had the greatest effect on quasi-extinction probability. For all four reintroduction strategies, the quasi-extinction probability decreased as the duration of the reintroduction effort increased. The change in quasi-extinction probability diminished at longer reintroduction durations; the decrease in quasi-extinction probability was minimal when the reintroduction period increased from 15 years to 20 years if the neonate survival rate in the wild was ≥ 0.30 (Fig 5). If the annual survival rate of neonates in the wild was ≥ 0.20, releasing juvenile snakes, adult snakes, or a mixed size-distribution all resulted in a lower quasi-extinction probability over a 30-year period than releasing neonates directly into the wild (Fig 5).

Including a large acclimation effect of captive-rearing on the survival of juvenile snakes changed the optimal release strategy under some conditions. When the annual neonate survival rate in the wild was 0.10 or 0.20, the survival rate of captive-reared juveniles during the first year post-release had to decrease by ≥ 50% to result in a higher quasi-extinction probability than releasing neonates directly into the wild (S3A and S3B Fig). When neonate survival was 0.30 or 0.40, direct release of neonate snakes only resulted in a lower quasi-extinction probability if the survival of captive reared juveniles post-release was reduced by ≥ 70% or 80% respectively (S3C and S3D Fig).

**Fig 6:**
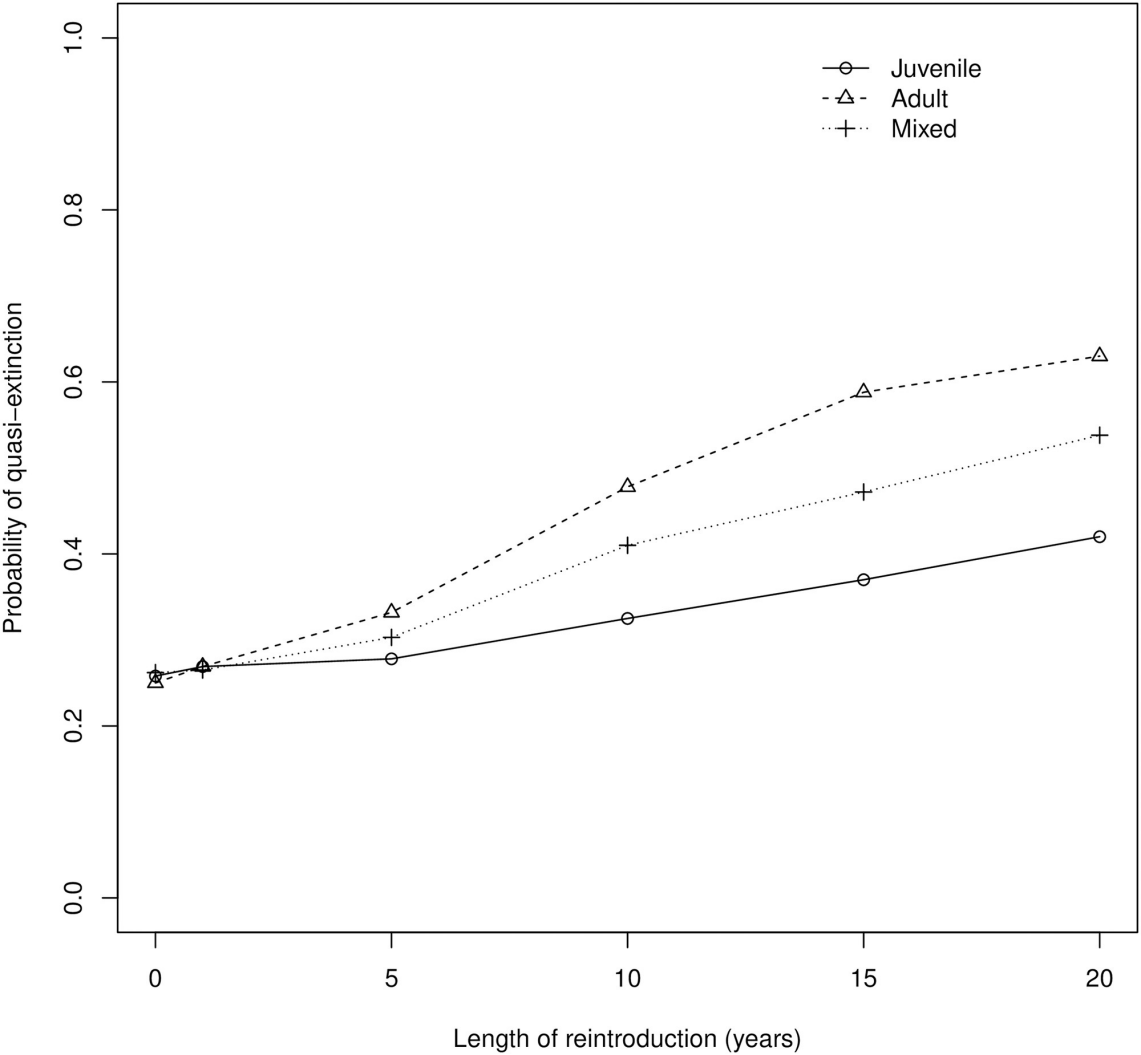
Probability of quasi-extinction (over a 30-year simulation) vs. the length of the reintroduction effort for donor populations of San Francisco gartersnake (*Thamnophis sirtalis tetrataenia*) with *N*_0_ = 100 females. For all simulations, the annual neonate survival rate in the wild was set to 0.30 and the number of adult female snakes required annually (either for captive-breeding or direct translocation) was 10. Strategies represent the release of captive-born juvenile snakes (B; circle and solid line), adult snakes translocated directly from the donor population to reintroduced population (C; triangle and dashed line), or a mixed size-distribution released (D; cross and dotted line).

Although releasing juvenile, adult, or a mixed size-distribution of snakes produced similar outcomes for the reintroduced population, these strategies had different effects on simulated donor populations. For all reintroduction strategies, the population initialized with N_0_ = 100 females (baseline quasi-extinction probability with no removals = 0.26) had a higher quasi-extinction probability than the population initialized with N_0_ = 650 females (baseline = 0.14). The longer the duration of the reintroduction, the higher the quasi-extinction probability for the donor population (Fig 6). A strategy based on releasing captive-bred neonate or juvenile snakes (while maintaining a captive population of 10 adult females) resulted in the lowest quasi-extinction probability for the donor population (1.5X increase relative to the baseline probability of quasi-extinction; Fig 6). Removing 10 adult snakes from the donor population to release in the reintroduced population on an annual basis had the highest quasi-extinction probability for the donor population (2.5X increase relative to baseline) and removing adult snakes for both direct release and captive breeding (mixed size-distribution of releases) had an intermediate quasi-extinction probability for the donor population (2.1X increase relative to baseline; Fig 6). In contrast, with *N*_0_ = 650 in the donor population, all reintroduction strategies resulted in a low probability of quasi-extinction and increasing reintroduction duration resulted in a minimal increase in the probability of quasi-extinction for the donor population (S4 Fig).

## Discussion

Reintroductions require substantial investment of time, effort, and money in an attempt to improve the prospects for species at high risk of extinction. Given the resources required and often perilous status of the species involved, it is imperative to employ strategies that give reintroduced populations the greatest potential viability. Our population simulations demonstrated that releasing head-started, 1-year-old juvenile San Francisco gartersnakes over a period of at least 15 years resulted in the lowest quasi-extinction probability for reintroduced populations. A reintroduction strategy based on the release of captive-born, head-started juvenile snakes also had the smallest effect on the viability of the donor population, compared to strategies that required more adult females to be removed from the wild. The survival and fecundity of adult females had the greatest influence on the population growth rate for San Francisco gartersnakes, based on an elasticity analysis of the IPM. Therefore, limiting the number of adult females removed from donor populations could be essential to ensure the few remaining San Francisco gartersnake populations are not negatively affected by reintroduction efforts.

The benefits of head-starting juvenile snakes come from increasing the survival rate of this vulnerable, early life stage relative to snakes in the wild. Size at birth is positively related to subsequent survival in some natricine snakes [37, 38] and survival of young neonate gartersnakes is lower than survival of larger, older gartersnakes in many populations [47, 54]. The survival rate of neonate San Francisco gartersnakes in the wild is unknown, but is likely lower than that of snakes ≥ 1 year old because smaller snakes are at greater risk of predation [55] and mortality during dispersal [56]. Although head-starting might increase the survival rate of neonate snakes, acclimation to captivity could result in altered phenotypes and decreased fitness in the wild post-release [57, 58]. For example, captive-reared eastern gartersnakes (*T*. *sirtalis sirtalis*) had smaller heads than wild conspecifics, which could limit the size of potential prey when released into the wild [59]. Decreases in fitness of translocated animals (release costs) might stem from maladaptive behavioral responses to the environment, increased movement rates, and naïve responses to potential predators [60–62]. Reptile reintroductions and translocations often result in reduced survival of released animals in the first few years post-release [63, 64]. For example, translocated adult giant gartersnakes (*T*. *gigas*) had greatly reduced survival compared resident snakes in the donor population [65]. Simulations therefore might overestimate the true rate of growth for reintroduced populations [66]. Given uncertainty about vital rates for reintroduced individuals, comparisons of the relative efficacy of reintroduction methods might be more reliable than absolute estimates of population viability [67].

Modeling can project potential outcomes before a reintroduction begins, but post-release monitoring is vital for assessing success. Using cover boards, drift fences and funnel traps to capture and mark individuals can provide data for capture-mark-recapture models to estimate the abundance [31, 33] and growth and survival [35] of San Francisco gartersnakes. If no San Francisco gartersnakes are present at the recipient site prior to reintroduction, recruitment can be estimated based on the capture of unmarked individuals in subsequent years [68]. Demographic monitoring alone will be insufficient to assess the viability of reintroduced populations, however. Modeling the effect of reintroduction strategies on genetic diversity could complement our demographic model, and genetic monitoring is crucial for quantifying genetic diversity and viability in reintroduced populations [69]. Genotyping all released snakes and new recruits could enable assessing which individuals are contributing to subsequent generations and estimate the amount of genetic variation in the new population [70, 71]. Although longer reintroductions had greater probability of success in our simulations, the chances of deleterious adaptations to captivity increase as the length of the captive breeding program increases [24]. Genetic monitoring can determine if adaptation to captivity is occurring [72] and whether mitigation needs to be taken to ensure releasing captive-bred animals does not negatively affect wild populations. The development of a reference genome for *T*. *sirtalis* from the California Conservation Genomics Project [73] could enable the estimation of adaptive genetic variation in captive and reintroduced populations [71].

A key next step will be to identify candidate sites with suitable aquatic and terrestrial habitat and sufficient prey for all life stages of San Francisco gartersnakes [74]. Further, if the reintroduction site was previously occupied, management might be required to ameliorate the factors that caused San Francisco gartersnakes to be extirpated. For example, reintroductions of native mammals in Australia were more likely to be successful if exotic mammal predators were absent or at low density [75]. Another valuable future step would be to evaluate the effectiveness of different release strategies, such as whether a “soft-release” in a wild enclosure increases snake survival relative to a hard release [62, 76]. For example, a recent review of snake translocation efforts found that several factors increased the odds of a positive result, including maintaining and releasing captive reared snakes in social groups and temporarily confining snakes at the release site [64]. A structured decision making (SDM) approach could be useful for identifying candidate sites for the release of translocated individuals [77]. A further benefit of using an SDM process would be to integrate the costs of reintroduction strategies into our analysis [19]. After reintroduction begins, SDM can be used in an adaptive management process to evaluate the success of initial efforts and alternative strategies that optimize multiple objectives [78]. Our model could be integrated into an SDM process and be updated as more data (e.g., better estimates of survival for wild neonates and juveniles) become available from the reintroduced population(s). The SDM process could also incorporate connectivity among extant and reintroduced populations of San Francisco gartersnakes, with the objective of increasing metapopulation viability through movement of individuals among populations.

## Conclusions

In the face of accelerating anthropogenic changes to biomes, reintroductions and translocations are increasingly used for the conservation of threatened and endangered species. Simulation studies using demographic models can identify optimal reintroduction strategies that are more likely to support viable reintroduced and donor populations. For the San Francisco gartersnake, reintroducing head-started juveniles was the strategy with the greatest chance of establishing a viable population, if snakes were released over a period of 15 or more years. Evaluating the efficacy of this strategy will require a robust monitoring plan to estimate the growth of the reintroduced population, and its genetic viability, in the long-term. Demographic models that simulate reintroductions could be integrated into an SDM process and be updated as more data are collected and project objectives are refined over time.

## Supporting information

S1 Fig Quasi-extinction vs. length of reintroduction with five adult females. Probability of quasi-extinction (over a 30-year simulation) vs. the length of the reintroduction (the number of years in which snakes are released) for reintroduced populations of San Francisco gartersnake (*Thamnophis sirtalis tetrataenia*) with a neonate survival rate in the wild of A) 0.10, B) 0.20, C) 0.30, or D) 0.40. The four lines in each plot correspond to the life-stage released into the reintroduced population, neonates, juveniles, adults, or mixed age/size-distribution. For all scenarios, five adult females are kept in captivity or released annually.
(TIF)

S2 Fig Quasi-extinction vs. length of reintroduction with three adult females. Probability of quasi-extinction (over a 30-year simulation) vs. the length of the reintroduction (the number of years in which snakes are released) for reintroduced populations of San Francisco gartersnake (*Thamnophis sirtalis tetrataenia*) with a neonate survival rate in the wild of A) 0.10, B) 0.20, C) 0.30, or D) 0.40. The four lines in each plot correspond to the life-stage released into the reintroduced population, neonates, juveniles, adults, or mixed age/size-distribution. For all scenarios, three adult females are kept in captivity or released annually.
(TIF)

S3 Fig Quasi-extinction and effect of acclimation to captivity. Probability of quasi-extinction (over a 30-year simulation) as a function of the effect of acclimation to captivity on survival of juvenile San Francisco gartersnakes (*Thamnophis sirtalis tetrataenia*) in the first-year post-release into the wild. A value of 0 on the x-axis represents no effect of captive-rearing on survival in the first-year post-release for juvenile snakes, a value of 0.9 represents a 90% decrease in survival in the first-year post-release for captive-bred juvenile snakes compared to the expected survival rate for juvenile snakes. Each black line represents reintroduction strategy B with the shape and line type indicating the duration of the reintroduction effort. Neonate survival rates in the wild were set to values of A) 0.10, B) 0.20, C) 0.30, or D) 0.40. Red lines indicate quasi-extinction probability for reintroduction strategy A (release of neonates to the wild shortly after birth) under varying durations of the reintroduction, for comparison to strategy B.
(TIF)

S4 Fig Quasi-extinction vs reintroduction effort for a donor population with *N*_*0*_ = 650. Probability of quasi-extinction (over a 30-year simulation) vs. the length of the reintroduction effort for donor populations of San Francisco gartersnake (*Thamnophis sirtalis tetrataenia*) with *N*_0_ = 650 females. For all simulations, the annual neonate survival rate in the wild was set to 0.30 and the number of adult female snakes required annually (either for captive-breeding or direct translocation) was 10. Strategies represent the release of captive-born juvenile snakes (B; circle and solid line), adult snakes translocated directly from the donor population (C; triangle and dashed line), or a mixed size-distribution (D; cross and dotted line).
(TIF)
