# Supplementary figures and images for: Comparing reintroduction strategies for the endangered San Francisco gartersnake (Thamnophis sirtalis tetrataenia) using demographic models
Source: PLoS One. 2023 Oct 5;18(10):e0292379. doi: 10.1371/journal.pone.0292379 (PMC10553336; doi:10.1371/journal.pone.0292379)

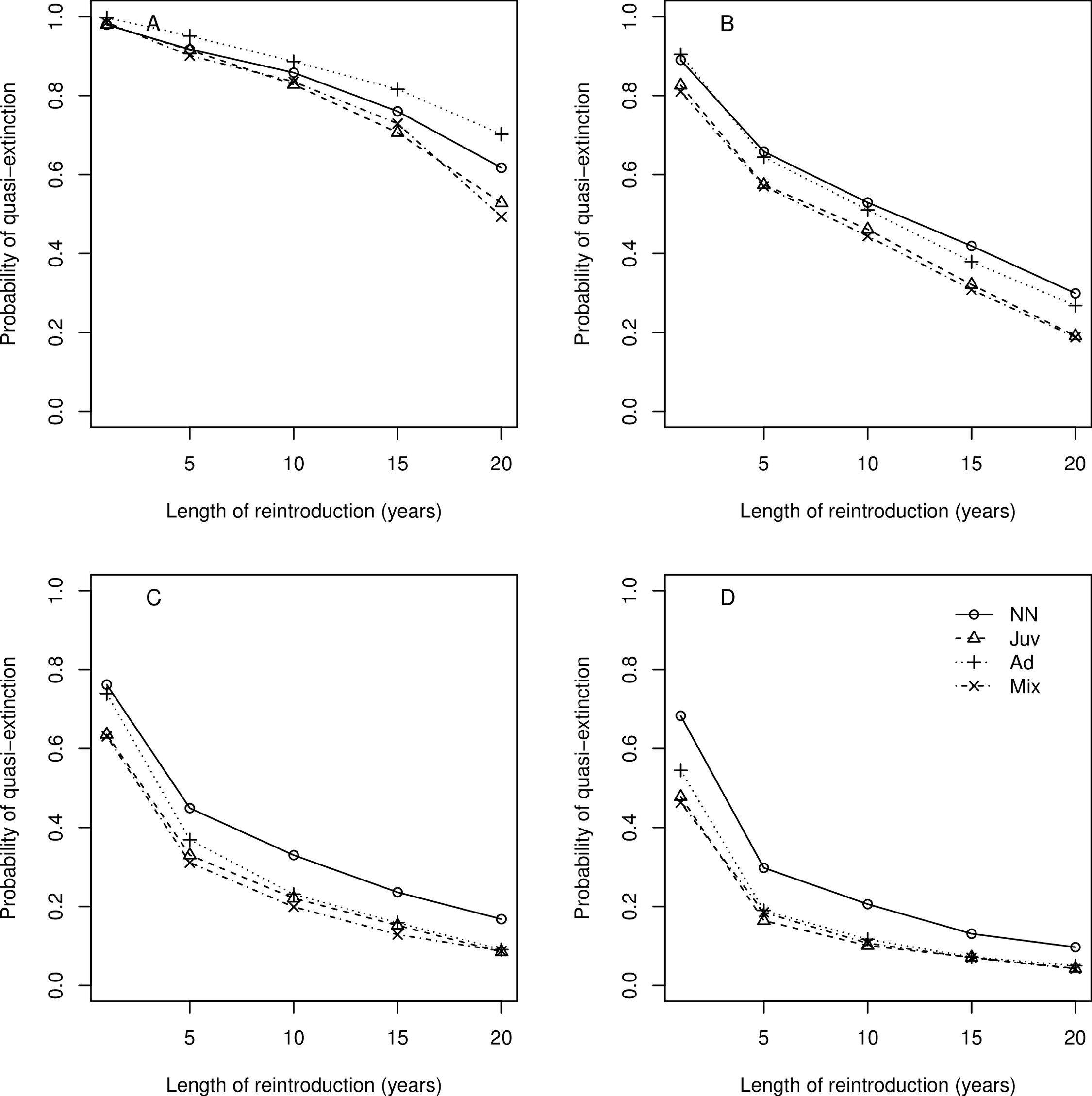

Supplement: S1 Fig — Probability of quasi-extinction (over a 30-year simulation) vs. the length of the reintroduction (the number of years in which snakes are released) for reintroduced populations of San Francisco gartersnake (Thamnophis sirtalis tetrataenia) with a neonate survival rate in the wild of A) 0.10, B) 0.20, C) 0.30, or D) 0.40. The four lines in each plot correspond to the life-stage released into the reintroduced population, neonates, juveniles, adults, or mixed age/size-distribution. For all scenarios, five adult females are kept in captivity or released annually. (TIF) [file pone.0292379.s001.tif]

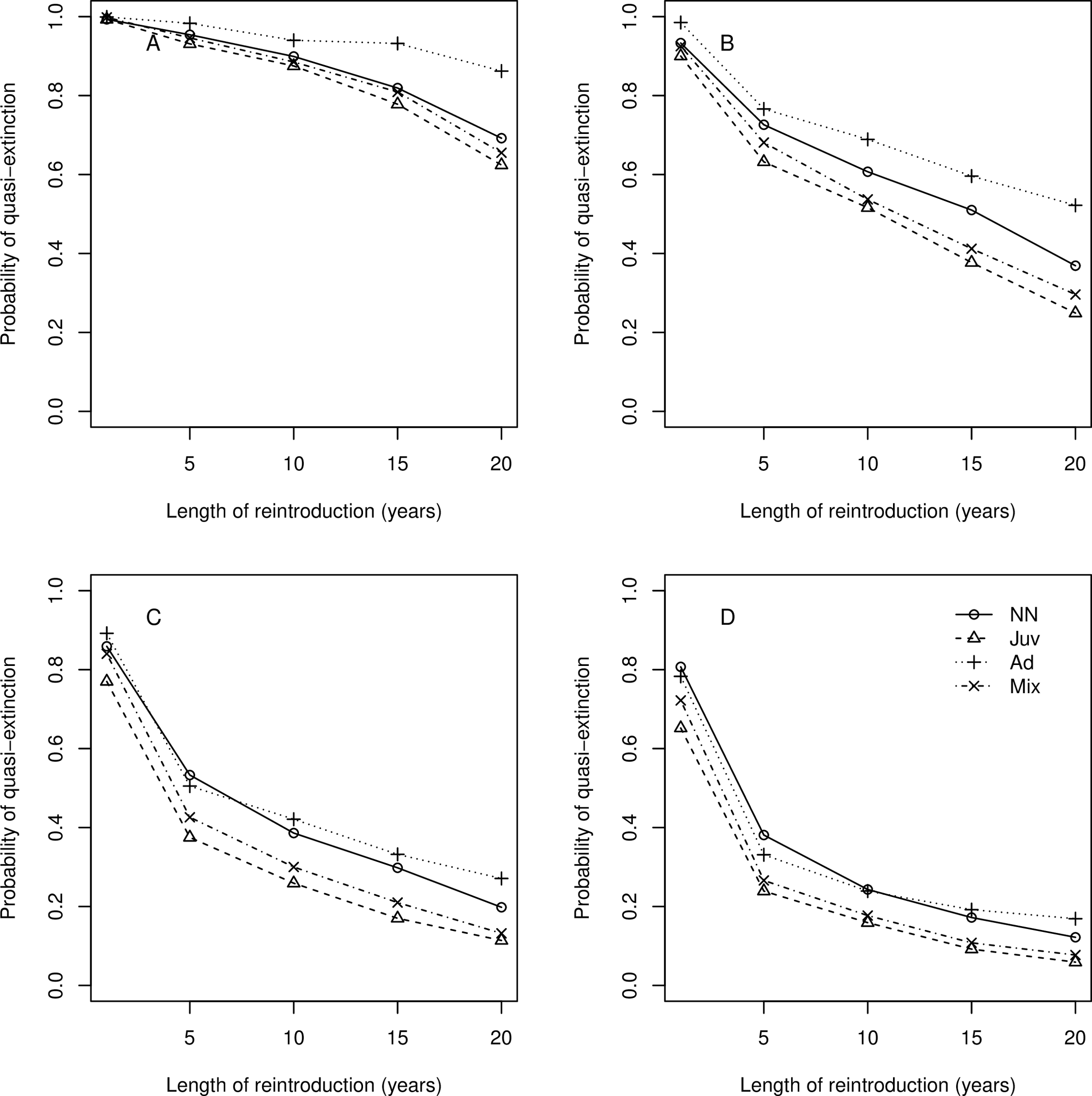

Supplement: S2 Fig — Probability of quasi-extinction (over a 30-year simulation) vs. the length of the reintroduction (the number of years in which snakes are released) for reintroduced populations of San Francisco gartersnake (Thamnophis sirtalis tetrataenia) with a neonate survival rate in the wild of A) 0.10, B) 0.20, C) 0.30, or D) 0.40. The four lines in each plot correspond to the life-stage released into the reintroduced population, neonates, juveniles, adults, or mixed age/size-distribution. For all scenarios, three adult females are kept in captivity or released annually. (TIF) [file pone.0292379.s002.tif]

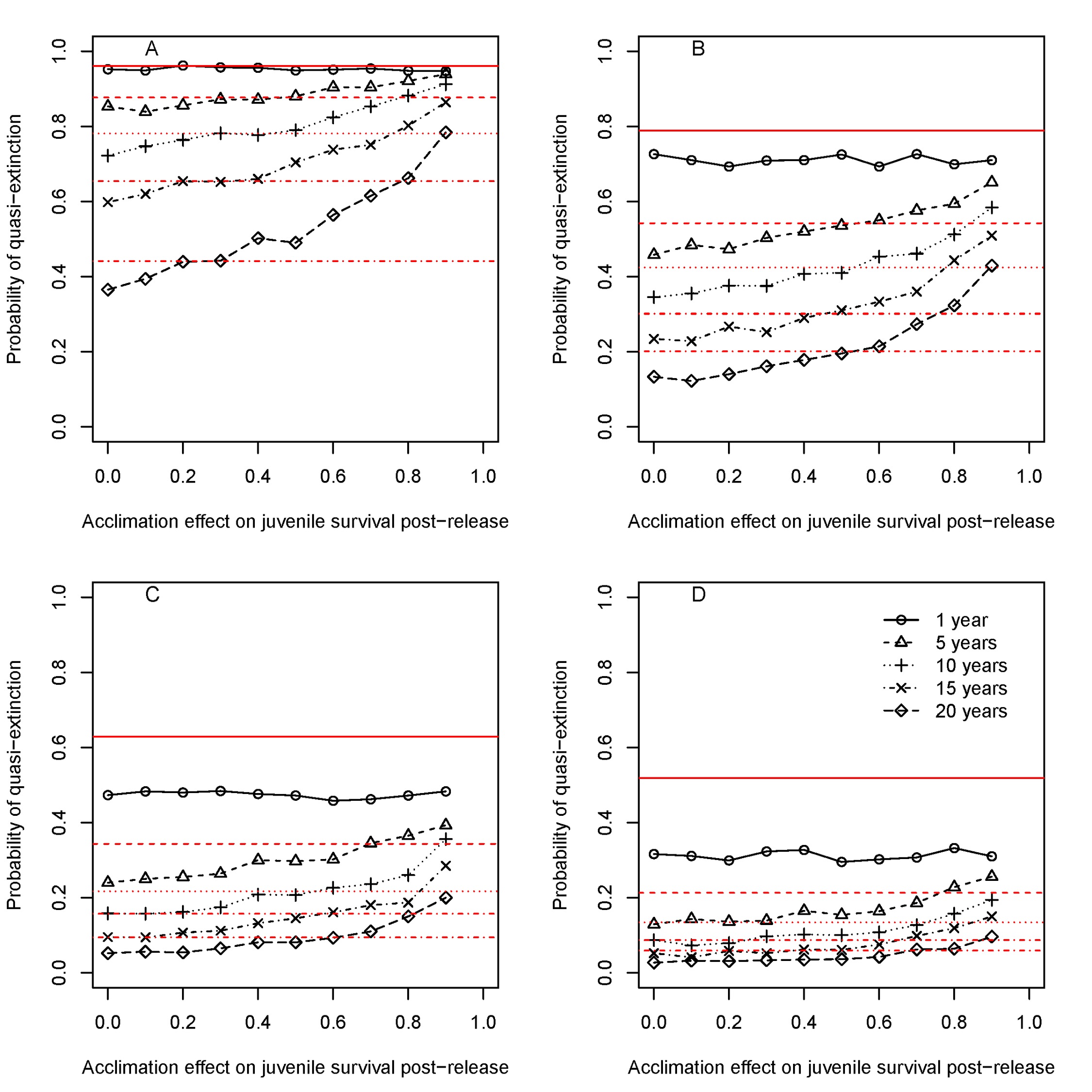

Supplement: S3 Fig — Probability of quasi-extinction (over a 30-year simulation) as a function of the effect of acclimation to captivity on survival of juvenile San Francisco gartersnakes (Thamnophis sirtalis tetrataenia) in the first-year post-release into the wild. A value of 0 on the x-axis represents no effect of captive-rearing on survival in the first-year post-release for juvenile snakes, a value of 0.9 represents a 90% decrease in survival in the first-year post-release for captive-bred juvenile snakes compared to the expected survival rate for juvenile snakes. Each black line represents reintroduction strategy B with the shape and line type indicating the duration of the reintroduction effort. Neonate survival rates in the wild were set to values of A) 0.10, B) 0.20, C) 0.30, or D) 0.40. Red lines indicate quasi-extinction probability for reintroduction strategy A (release of neonates to the wild shortly after birth) under varying durations of the reintroduction, for comparison to strategy B. (TIF) [file pone.0292379.s003.tif]

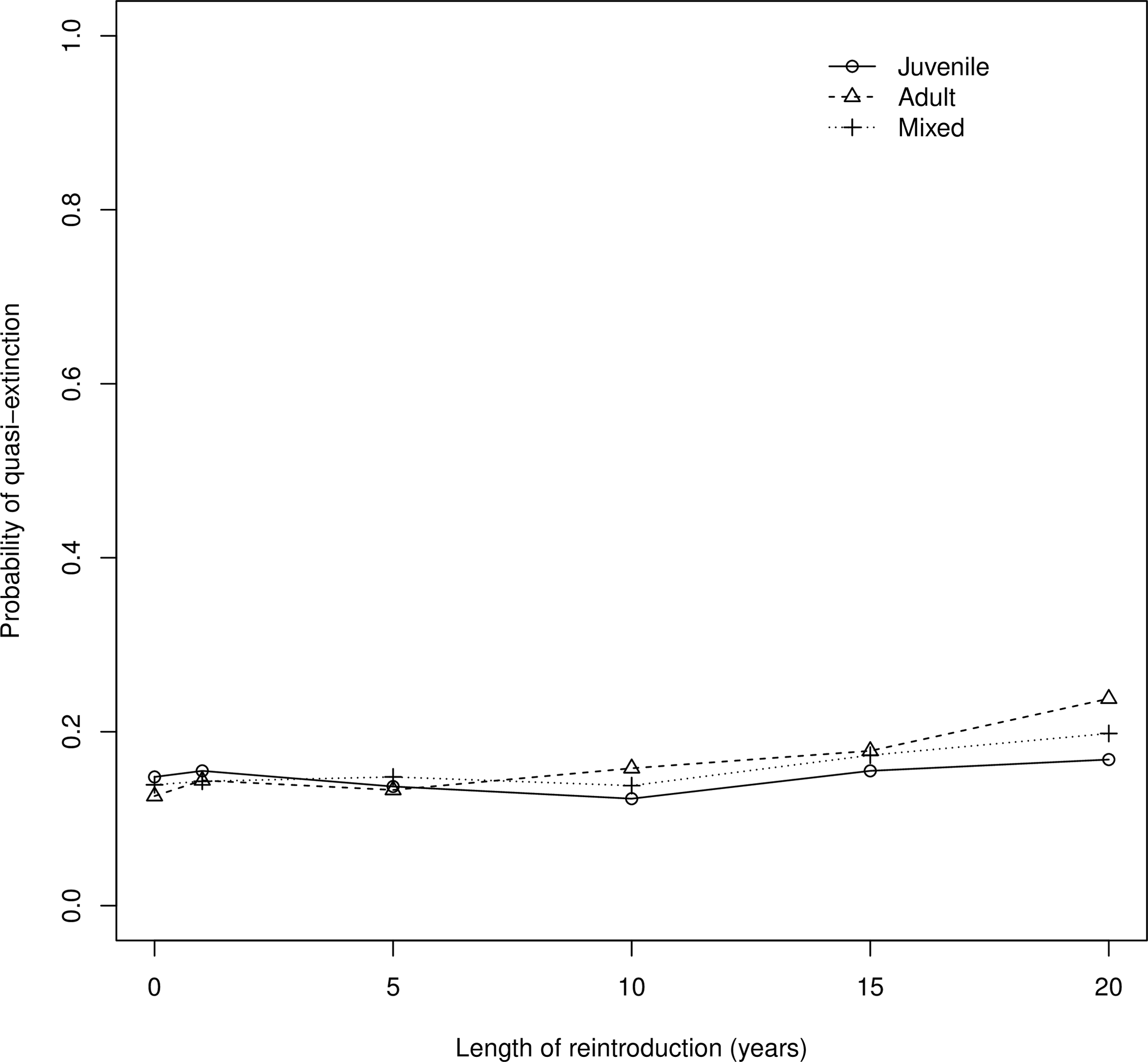

Supplement: S4 Fig — Probability of quasi-extinction (over a 30-year simulation) vs. the length of the reintroduction effort for donor populations of San Francisco gartersnake (Thamnophis sirtalis tetrataenia) with N0 = 650 females. For all simulations, the annual neonate survival rate in the wild was set to 0.30 and the number of adult female snakes required annually (either for captive-breeding or direct translocation) was 10. Strategies represent the release of captive-born juvenile snakes (B; circle and solid line), adult snakes translocated directly from the donor population (C; triangle and dashed line), or a mixed size-distribution (D; cross and dotted line). (TIF) [file pone.0292379.s004.tif]
